# Don’t jump the gun quite yet: aiming for the true target in plant neurobiology research

**DOI:** 10.1007/s00709-024-01993-4

**Published:** 2024-09-28

**Authors:** Paco Calvo, Vicente Raja, Miguel Segundo-Ortin

**Affiliations:** Minimal Intelligence Laboratory (MINT Lab), University of Murcia, Murcia, Spain

**Keywords:** Plant Intelligence, Plant Neurobiology, Historical Evidence, Bias, Adversarial Collaboration, Scientific Discourse

## Abstract

In their recent paper, Kingsland and Taiz argue that proponents of plant intelligence and plant neurobiology misuse historical sources to support their claims, suggesting a pattern of bias. They critique the reliance on subjective judgments and the systematic misuse of past literature by notable scientists. This response addresses their criticisms while adhering to Rapoport’s rules to foster constructive academic dialogue. We emphasize the importance of evidence-based research and highlight areas of agreement, including the fallacy of appealing to authority and the necessity for more robust empirical evidence. However, we also challenge their selective citation practices and argue that their narrative itself is subject to the same criticisms they levy. By examining recent works and pointing out overlooked rebuttals, we aim to clarify misconceptions and advocate for a more nuanced understanding of plant intelligence research. This dialogue underscores the need for rigorous, respectful scientific discourse to advance the field.

In their recent paper “Plant ‘Intelligence’ and the Misuse of Historical Sources as Evidence,” Kingsland and Taiz contend that advocates of plant intelligence (and plant neurobiology) often misuse historical sources to support their claims. The authors argue that this practice reveals a potential pattern of bias among proponents of plant intelligence; a pattern that drives them to support preconceived ideas rather than critically analyzing them in their historical context. In their view, not only does the concept of plant intelligence rely far too heavily on subjective judgments and lack scientific validation, but defenders of the view have also systematically misused past literature by authors such as Darwin, Sachs, Went, Thimann, McClintock, and Lamarck as alleged sources of historical evidence.

Before we delve into the specifics of the critique and our response, a caveat is in order. We thank our critics for their thoughtful and sustained critique of our research on plant intelligence and for the time and effort they have devoted throughout the years to this endeavor (Draguhn et al. 2021; Mallatt et al. 2021a; 2021b; 2021c; 2023; Robinson and Draguhn 2021; Robinson et al. 2023; 2024; Taiz et al. 2019; 2020). We appreciate the opportunity to engage in this dialogue and respond to the critiques.

However, we urge all authors to avoid *ad hominem* attacks, as such remarks detract from constructive debate. Unfortunately, this is not the first time we have encountered such remarks in print. In a previous article, Mallatt et al. (2021a) concluded with the following set of ideas:

Our 12 counterarguments are important to the future of plant biology, because dubious ideas about plant consciousness can harm this scientific discipline. We foresee three types of harm. First, not only does the notion of plant consciousness mislead the general public, but it also can generate mistaken ideas about the plant sciences in young, *aspiring plant biologists*. Second, the strong, romantic appeal of plant consciousness could influence public and private *funding agencies* to fund projects that are based on its fallacies. Third, public acceptance of plant consciousness could affect research *regulation*. For instance, could research on genetically modified plants face even more resistance if plants were regarded as conscious? How might laboratory-research regulations be impacted when scientists are seen to per- form invasive manipulations on plants that feel pain?

These are not idle concerns. Articles that promote plant neurobiology thinking are increasingly finding their way into respectable scientific journals—even top-tier journals (Calvo and Friston 2017; Tang and Marshall 2018; Baluška and Manusco 2020; Calvo et al. 2020). This is most regrettable, and hopefully our article, by putting the record straight, will reverse this trend. In conclusion, we feel we must speak forcefully: plant neurobiologists have become serial speculationists. The ratio of speculation to data in their oeuvre is astronomically high. If they want to form a sensible hypothesis and then test it with real experiments, that is fine, but the prolific speculating and fantasizing need to stop. (Mallatt et al. 2021a)

These remarks are unfortunate. We do *not* believe that our opponents’ ideas harm the field. Quite the contrary, we believe in the value of adversarial collaboration (Clark et al. 2022). The field is harmed when it is suggested that opposing views “mislead the general public.” We also do *not* believe that our opponents’ views represent a threat to “young, aspiring plant biologists.” Disagreeing with our critics does not lead us to think that their research should not be funded (“… public and private funding agencies to fund projects that are based on its fallacies.”). Do they mean to imply that our research is not respectable when they state: “Articles that promote plant neurobiology thinking are increasingly finding their way into respectable scientific journals”? And who is supposed to put the record straight when they claim: “… our article, by putting the record straight, will reverse this trend”? Which members of the scientific community can arrogate to themselves the intellectual authority to put the record straight? We believe the answer is so obvious that it does not need to be stated. We encourage rigorous academic debate and constructive criticism, but this must comply with common ethical standards and professional courtesy.

It is a fundamental principle of scholarly discourse that critiques should focus on the ideas, methods, and findings presented in the work, rather than on the individuals who conducted the research. Ending an article with expressions such as “the prolific speculating and fantasizing need to stop” does a disservice to the field. We do not believe that researchers with opposing views to ours are fantasizing. The good news about Science (with a capital S) is that all we need to do is submit our respective working hypotheses to empirical scrutiny. Then, time will tell. But please, no vetoes raised in academic journals.

Finally, it is important to clarify that we speak only for ourselves, not for all proponents of plant intelligence or plant neurobiology. The field includes diverse perspectives, and researchers’ motivations vary widely. Not everyone who supports plant intelligence aligns with plant neurobiology, and motivations differ. Thus, our response addresses Kingsland and Taiz’s criticisms of our work, representing our views and practices, not the entire scholarly community.

Enough of the preliminaries: In the spirit of fostering constructive dialogue, we draw upon *Rapoport’s rules* (1960; 1961), as articulated by Daniel Dennett in his *Intuition Pumps and Other Tools for Thinking*. Dennett recommends an approach to criticism that serves as the best antidote to the common “tendency to caricature one’s opponent” (p. 33). He advises that, before offering any rebuttal or criticism, you should:

1. … attempt to re-express your target’s position so clearly, vividly, and fairly that your target says, “Thanks, I wish I’d thought of putting it that way.”
2. … list any points of agreement (especially if they are not matters of general or widespread agreement).
3. … mention anything you have learned from your target.
4. Only then are you permitted to say so much as a word of rebuttal or criticism. (Dennett 2013, pp. 33–4).

We believe that adhering to these guidelines will enable us to address Kingsland and Taiz’s critique in a manner that is both respectful and productive. To this end, we will first highlight areas of concurrence and recognize insights gained from their perspective.

## Points of agreement

The following six points of agreement are worth noting:

- #1. Appealing to authority is a fallacious move. Kingsland and Taiz observe that:

Overall, the common practice by proponents of plant intelligence and plant consciousness of uncritically citing the words of eminent scientists of the past, taken out of their historical context to bolster their arguments, should not be confused with scientific evidence supporting these concepts, even when the quotations, themselves, are accurate.

- #2. *The evidence, the evidence, and nothing but the evidence*. Throughout their article, Kingsland and Taiz insist that the scientific evidence in our favor is lacking:

… criticisms to date have focused on whether proponents of these views have been able to marshal clear scientific evidence in support of their arguments…

The criticisms addressed several recurring problems, including the lack of evidence for extraordinary claims…

A critique of a theory about the cellular basis of consciousness concluded that “to become accepted, theories require proof from hypotheses-testing, solid facts and empirical evidence” and all these things were lacking from the cellular consciousness theory…

- #3. *Speculative status of current research*. Commenting on *Planta Sapiens*, Calvo’s popular book (with Natalie Lawrence), Kingsland and Taiz observe:

Throughout these two decades, claims for plant intelligence and related ideas remained speculative. In 2022, Calvo and coauthor Natalie Lawrence could only propose that “we suspect that plants think,” while also pointing out that they did not yet know “how they think,” and argued that more science was needed to “see behind their seraphic, sphinx-like pose” (Calvo and Lawrence 2023, p. 131).

Three additional points of agreement are in order before we proceed with the rebuttals:

- #4. *Of cults and popular books*. Kingsland and Taiz rightly warn readers against the dangers of cults fueled by popular books of dubious scientific value:

Current proposals for a new science of “plant intelligence” differs from the controversy generated by Peter Tompkins and Christopher Bird’s book, *The Secret Life of Plants*, in 1973 (Tompkins and Bird 1973). Neither author was a scientist, and the book drew on dubious electrophysiological polygraph experiments on plants to promote the notion that plants were intelligent, reacted to human speech, and even answered back in plant language. One reviewer described the book as a popular-science pastiche of New Occult hopes (Pace 1996). In the 1970s, scientists debunked these claims as fallacious and unprovable (Galston 1974), but found to their dismay that they were “being kept alive in the popular literature by highly efficient mass-media techniques” (Galston and Slayman 1979, p. 344).

- #5. *The threat of humanizing plants*. In like vein, Kingsland and Taiz are correct in raising the concern that:

Attempts to humanize trees, or to argue that we should care about trees because they are like people, could mislead the public about what kinds of solutions will be needed to preserve the resilience of forest ecosystems (Robinson et al. 2023).

- #6. *On misreferenced quotations*: In commenting on Anthony Trewavas’ 2014 book, *Plant Behavior and Intelligence*, Kingsland and Taiz note that:

The second chapter of his book begins with an epigraph purporting to be from Darwin: “Intelligence is based on how efficient a species becomes at doing the things they need to survive.” (Trewavas 2014, 10). It is dated 1871, implying it comes from Darwin’s *Descent of Man*, although *Descent* is not listed as a source. However, the same quotation is used in a later article published by Paco Calvo, Monica Gagliano, Gustavo Souza, and Trewavas in 2020, and there the bibliography identifies the source as *Descent of Man* (Calvo et al. 2020).

We could not agree more with #1–#6: the proof is in the pudding. What counts is the scientific evidence itself (together with logical reasoning), regardless of who held what and when. Doing otherwise would be to fall prey to an “argument from authority” (an argumentative fallacy). We hope their own quotation from *Planta Sapiens* serves to illustrate clearly how much we are on the same page concerning the evidential basis for research, and with regard to speculation in science.

In the preface to their popular book, Calvo and Lawrence warn that:

Caution is necessary: whether you are deeply sceptical of the possibility that plants might have intelligence or are an enthusiastic believer in the supernatural wisdom of other lifeforms, we all need to broaden our minds carefully. To dramatically shift our understanding of the world in a measured way, based on the evidence as it emerges. I neither want to narrow-mindedly ignore the astounding possibilities of what science is uncovering nor to start a new animistic cult of nature worship.

We are confident that this excerpt further illustrates the points of agreement with our critics.

Lastly, with respect to #6, we could not agree more with the need to be extra careful when referencing and quoting. Unfortunately, unintentional mistakes can and do occur. As humans, we all err. In this particular case, the misquoted statement, having not been noticed, was carried into further publications, inheriting the “original sin.” We cannot change the past, but we can avoid using the fake quote in future work.

In the spirit of Rapoport’s rules, we trust points #1–#6 offer sufficient agreement and hope we can now offer some constructive criticism.

## Words of rebuttal

Despite these agreements, many disagreements remain.

While we agree that arguments from authority should be avoided, it is noteworthy that our critics rely more on the credibility of certain scientists than on logical reasoning and empirical evidence. If not an *appeal to authority*, why devote five lines to listing coauthors and their affiliations?:

The growth of the field of “plant neurobiology” between 2003 and 2006 provoked a strong critique in 2007 signed by an international group of 36 biologists representing 33 academic institutions located in the USA, Canada, U.K., Germany, Italy, Switzerland, France, and the Netherlands (Alpi et al. 2007). The article was drafted by David G. Robinson, a cell biologist at the University of Heidelberg, and at his invitation, 35 other scientists agreed to be coauthors.

Why not simply say “Alpi et al.”? If they are correct, it is not because they are globally affiliated. Would it add credibility to plant neurobiology if one of us had a solo piece signed by global sympathizers? This could be done easily, yet we suspect our critics would accuse us of using “speculators and fantasizers.” While expertise can lend credibility, it is the evidence and reasoning that matter, not who makes the claim. Let us avoid double standards.

After previously criticizing the lack of empirical evidence in a series of articles, Kingsland and Taiz now focus on historical evidence. In their own words,

we now wish to turn to the way this community has used, and continues to use, historical authorities as *sources of evidence*. Such uses are meant to provide authoritative “evidence” in favor of their approach, and we should accordingly look closely at how this evidence is presented.

And they complain about the way historical evidence has been presented in the plant intelligence/neurobiology literature in the past:

The habit of **doctoring **quotations in seemingly slight but significant ways helps to **slant** the argument in favor of plant intelligence and plant neurobiology. In the case of J. C. Bose as forerunner, one explanation offered for the neglect of Bose hinges on the charge of racism, but ignores evidence that scientists had well-founded reservations about Bose’s interpretations and experimental results. In all these cases historical sources are being used instrumentally in support of foregone conclusions.

[…] The repeated use of these quotations means that misleading claims are repeated over many years and are never corrected. Most reviewers, referees, and readers will not be familiar with the historical sources, will not check them for context or accuracy, and may not realize that a **highly distorted picture of past scientific practice is being presented as fact**. Readers with little historical background are unlikely to appreciate the level of distortion that is rampant in this literature. […] Once the distortion is revealed, the value of such “evidence” as support for ideas about plant intelligence and related concepts evaporates. That an argument in support of a *scientific* field has used such a constant level of **factual distortion** should be troubling, and should cause us to reflect on how such things can be allowed to occur. (emphasis ours. See discussion below)

Before addressing the more concerning aspect of Kingsland and Taiz’s critique (note terms in bold), it is useful to note that their narrative faces the same criticism they raise. They focus on recent history (2002–2024) rather than historical figures. While we acknowledge factual mistakes can occur, we take issue with the pattern of distortion they seem to apply in portraying the recent history of the field.

Their article is filled with such *patterns*, though we cannot address them all here. For illustration, consider the following two excerpts, with our responses, which seem to present, borrowing from them, “a highly distorted picture of past [in this case, contemporary] scientific practice”:

These discussions attracted the attention of a philosopher of science, Paco Calvo, who wrote a manifesto proposing that the philosophy of plant neurobiology was an emerging field at the intersection of plant neurobiology and philosophy of cognitive science (Calvo 2016). In 2014 Trewavas published his own manifesto in a book, *Plant Behaviour and Intelligence* (Trewavas 2014; Lev-Yadun 2015). A stream of popular books supported this enterprise and broadened its audience (Mancuso and Viola 2015; Wohlleben 2016; Mancuso 2018; Gagliano 2018; Ryan et al. 2021; Calvo and Lawrence 2023). Suzanne Simard, a forest scientist and ecologist, extended these ideas to include mycorrhizae, positing that the networks created through these symbiotic relationships were similar to the neural networks of animals, and therefore could give rise to a form of intelligence (Simard 2018; 2022). In 2024 journalist Zoë Schlanger published a sympathetic profile of the plant intelligence community, as well as of other scientists investigating plant adaptations (Schlanger 2024).

In effect, in 2016, Calvo presented a “philosophy of” plant neurobiology manifesto, emphasizing its role as a metadiscipline—similar to the philosophy of biology, chemistry, or physics. This field, emerging at the intersection of plant neurobiology and cognitive science, seems overlooked in their skepticism. As Calvo and Lawrence noted in *Planta Sapiens*: “Physiology needs psychology. As the prominent American psychologist Edward C. Tolman wrote in the mid twentieth century: ‘A psychology cannot be explained by a physiology until one has a psychology to explain.’” The key point is that psychology is essential to understanding, not whether Tolman highlighted this decades ago.

This is a point our critics often miss: studying animal intelligence falls under cognitive science, not animal physiology. Animal physiology focuses on functions and mechanisms; on biological processes such as homeostasis, respiration, circulation, and muscle function, to name but a few (Hill et al. 2012). Research on animal intelligence is conducted within cognitive science, specifically in comparative cognition, which combines insights from psychology, neuroscience, ethology, and other fields to understand the mental processes underlying animal behavior (e.g., learning and memory, problem-solving, and communication, among others). Similarly, plant intelligence is not fully addressed by plant physiology alone, which examines the physical and biochemical processes within plants, such as photosynthesis, nutrient uptake, and growth regulation. While these are crucial for understanding how plants function, they do not encompass the study of plant behaviors that might be considered “intelligent,” such as decision-making, memory, and flexible and anticipatory responses. Just as cognitive science provides the framework for studying animal intelligence, a similar transdisciplinary approach is required for studying plant intelligence. This field blends plant physiology with concepts from cognitive science, ecology, and, yes, even philosophy; hence the need for a philosophy of plant neurobiology manifesto.

Kingsland and Taiz do not seem to understand that our focuses diverge: theirs on physiological functions and mechanisms, ours on behavior and cognition. Instead, they inadvertently suggest an orchestrated movement within the plant intelligence community by listing various authors and works in a single paragraph. This name-dropping creates an illusion of coordination, misrepresenting independent scholarly contributions. Many of these authors likely have not read each other’s books, collaborated, or even met, which undermines the idea of a unified agenda.

This approach ironically mirrors their own criticism of distorting historical facts. By lumping diverse works together, they appear to imply that if one book is pseudoscientific, all are discredited, ignoring the independent nature of each contribution. This portrayal is misleading and detracts from a fair evaluation of the field’s scientific merits. Citing titles like *Brilliant Green*, *The Hidden Life of Trees*, *The Mind of Plants*, *Planta Sapiens*, *Finding the Mother Tree*, and *The Light Eaters* in a single paragraph, as if these works belong to a coordinated effort, misleads readers into inferring a singular, orchestrated movement.

Not only do Kingsland and Taiz fail to appreciate their own pattern of distortion, but they also continue:

Throughout these two decades, claims for plant intelligence and related ideas remained speculative. In 2022, Calvo and coauthor Natalie Lawrence could only propose that “we suspect that plants think,” while also pointing out that they did not yet know “how they think,” and argued that more science was needed to “see behind their seraphic, sphinx-like pose” (Calvo and Lawrence 2023, 131). Their proposals, however, depended heavily on viewing plants as being like animals: the book was filled with examples taken from studies of animal behavior, followed by the suggestion that plants might be thought to behave similarly. Despite this heavy reliance on analogy and speculation, the discourse on plant intelligence and related concepts has expanded its reach and become a cultural phenomenon of wide-reaching impact.

We have repeatedly addressed the misunderstanding of “analogy and speculation” in previous work (Calvo and Trewavas 2020; Calvo and Lawrence 2023; Segundo-Ortin and Calvo 2023). More troubling is that we clarified this point in a detailed reply in the journal *Animal Sentience*, responding to the same group of authors. The fact that Kingsland and Taiz did not cite our previous response (Calvo and Segundo-Ortin 2023) is symptomatic of the ongoing pattern of distortion they seem to engage in.

As we explained in Calvo and Segundo-Ortin (2023), in the context of the scientific study of plant sentience:

We find the accusation of anthropomorphism puzzling. What is the basis for this charge? Unless we assume that sentience is a capacity that belongs only to human beings, we fail to see how considering the possibility that plants have felt states reflects an anthropomorphic view of plants. Nowadays, more and more non-human animal species are being considered sentient (and probably rightly so!) and none of these conclusions are regarded as anthropomorphic. By the same token, we do not see why the hypothesis of plant sentience implies this bias.

The charge of zoocentrism is a long-standing one. It sometimes concerns the methods being used to investigate plant behavior. For instance, in the past, some of our commentators have insisted that time-lapsing plants (one of the most valuable tools in studying plant behaviour) serves “to make them look more animal-like” (Taiz et al. 2019, p. 684).

The criticism that the use of time-lapse videos zoomorphizes plants is misplaced. Time- lapse techniques are not used to make plants appear "more animal-like" but to uncover intricate patterns in plant behavior that might otherwise go unnoticed to the naked eye (Calvo and Trewavas 2020). These methods provide valuable data on various aspects of plant movement.

We could elaborate further, but we will spare the details (see Calvo and Segundo-Ortin (2023) for a response to Mallatt et al. (2023) and Robinson et al. (2023), both coauthored by Taiz). For now, considering Kingsland and Taiz issues with the alleged “doctoring” of historical sources “to slant” our arguments in one direction, what should we make of their own slant? Is selective quotation and ignoring published literature not a form of distortion? And if it is, are they not using their “evidence” to support their ideas? Why ignore research by neuroscientists (not philosophers) that calls for a “broadening of the definition of a nervous system to better understand the evolution of plants and animals” (Miguel-Tomé and Llinás, 2021)? Why would not this pattern of distortion in their narrative be seen as a bias, as they claim in our case? Let us not use double standards.

The key difference between our cases is that, while they openly discuss doctoring or slanting arguments, we do not suggest they have manipulated recent history to suit their goals, or slanted their coverage intentionally.

Instead, in the spirit of Rapoport’s rules, we choose to be charitable in our interpretation. Perhaps they have not read our response (Calvo and Segundo-Ortin 2023), even though it was published before they submitted their paper to *Protoplasma*. Or perhaps they read it but did not find it worth responding to or citing. The same applies to the other works they have overlooked, or their selective quoting from *Planta Sapiens*.

A clear example is our previous work (Ponkshe et al. 2023), where we discuss the challenges in replicating Gagliano et al.’s (2016) pea plant associative learning study. Why is this not included in their review of the field? Or consider our analysis of how a pole affects bean shoot nutation patterns (Raja et al. 2020). Contrary to our critics’ claim (Mallatt et al. 2023), our approach does not overlook the causal mechanisms behind bean behavior. In Raja et al. (2020), we explain, using jargon devoid of any “cognitive” load whatsoever, that:

our research introduced a cutting-edge methodology to study the dynamics of plant nutation movements. Unlike traditional kinematic analyses focused on shape, period, or amplitude, our approach centred on three distinct aspects that are widely recognized in the biological and behavioural dynamics literature as signatures of adaptively controlled processes and motions when found in other organisms: harmonicity, predictability, and complexity.

Our point was flagged in our response to Mallatt et al. (2023). In fact, as we stated in the abstract of Calvo and Segundo-Ortin (2023):

we proposed the intriguing possibility of plant sentience, drawing parallels with non-human animal studies. This response aims to sift through the rich thicket of perspectives offered by our commentators. To do so, we assess the risks of employing double standards, as well as the tendencies of anthropomorphizing and zoomorphizing in plant studies. We also emphasize the need for clarity in linguistic and conceptual terms, examine the neurophysiological evidence for plant sentience, and discuss the ethical implications of such recognition.

Despite addressing many of Taiz and collaborators’ concerns, why do Kingsland and Taiz fail to cite Raja et al. (2020) and Calvo and Segundo-Ortin (2023)? Instead, they cluster unrelated works together. Being charitable, we conclude this is not intentional distortion. Yet, if we were not charitable, it might seem they ignore our rebuttals and research with negative or inconclusive results to misrepresent the field and our arguments. This oversight not only perpetuates misrepresentation but also undermines academic discourse by failing to acknowledge readily available counterarguments. However, we are not asserting this conclusion.

Probably, the best way to portray the views of some of us is reflected in the acknowledgments of *Planta Sapiens*, which speak directly to “the detractors of mine and my colleagues’ work”:

Of course, there can be no progress in any field without tension and disagreements. Sometimes these are fierce, I’m afraid. But, despite that, I’m grateful to have had the chance to test my ideas and work in my interactions with Lincoln Taiz, Michael Blatt and David Robinson. Criticism and opposition can only have made me work harder. If they happen to read this book at all, I hope they might consider rethinking some of their criticisms. (Calvo and Lawrence 2023)

Much more could be said in response to Kingsland and Taiz, but first things first: our critics are encouraged to follow Rapoport’s rules. We cannot resist quoting some of Dennett’s remarks from his introduction to Rapoport’s rules. Dennett observes:

But the search for hidden contradictions often crosses the line into nitpicking, sea-lawyering, and—as we have seen—outright parody. The thrill of the chase and the conviction that your opponent *has* to be harboring a confusion somewhere encourages uncharitable interpretation, which gives you an easy target to attack. But such easy targets are typically irrelevant to the real issues at stake and simply waste everybody’s time and patience, even if they give amusement to your supporters (Dennett, p. 33).

Please remember to try to re-express your target’s position fairly, identify points of agreement, and learn something from them. Only then should you be allowed “to say so much as a word of rebuttal or criticism” (Dennett 2013, pp. 33–34). In the spirit of fostering constructive academic dialogue, we acknowledge that Kingsland and Taiz’s critique centers on concerns about the misuse of historical evidence and the importance of rigorous standards in scientific discourse. By recognizing their emphasis on ensuring accurate and responsible use of historical sources, we have been reminded of the need for meticulous scrutiny in both historical and experimental contexts. Their challenges have pushed us to refine our approaches, reinforcing that such critical engagement is crucial for the advancement of any scientific field, including plant neurobiology. For these reasons, we are grateful for the dialogue, which we hope contributes to a deeper understanding of the diverse perspectives within this emerging field.

In the meantime, don’t jump the gun.

## References

[CR1] Alpi A, Amrhein N, Bertl A et al (2007) Plant neurobiology: no brain, no gain? Trends Plant Sci 12:135–136. 10.1016/j.tplants.2007.03.002 10.1016/j.tplants.2007.03.002 17368081

[CR2] Baluška F, Manusco S (2020) Plants, climate and humans. EMBO Reports e50109. 10.15252/embr.202050109 10.15252/embr.202050109 PMC7054678 32103598

[CR3] Calvo P (2016) The philosophy of plant neurobiology: a mani-festo. Synthese 193:1323–1343. 10.1007/s11229-016-1040-1

[CR4] Calvo P, Friston K (2017) Predicting green: really radical (plant) predic-tive processing. J R Soc Interface 14:20170096. 10.1098/rsif.2017.0096 10.1098/rsif.2017.0096 PMC5493793 28637913

[CR5] Calvo P, Lawrence N (2023) Planta sapiens: the new science of plant intelligence. W. W. Norton & Company, New York

[CR6] Calvo P, Segundo-Ortin M (2023) Plant sentience revisited: sifting through the thicket of perspectives. An Sent 8(33):32. 10.51291/2377-7478.1830

[CR7] Calvo P, Trewavas A (2020) Physiology and the (neuro)biology of plant behavior: a farewell to arms. Trends Plant Sci 25:214–216. 10.1016/j.tplants.2019.12.016 10.1016/j.tplants.2019.12.016 31926766

[CR8] Calvo P, Gagliano M, Souza GM, Trewavas A (2020) Plants are intelli-gent, here’s how. Ann Bot 125:11–28. 10.1093/aob/mcz155 10.1093/aob/mcz155 PMC6948212 31563953

[CR9] Clark CJ, Costello T, Mitchell G, Tetlock PE (2022) The road less travelled: understanding adversaries is hard but smarter than ignoring them. J Appl Res Mem Cogn 11:50–53. 10.1037/mac0000020

[CR10] Dennett DC (2013) Intuition pumps and other tools for thinking. Norton & Company, New York

[CR11] Draguhn A, Mallatt JM, Robinson DG (2021) Anesthetics and plants: no pain, no brain, and therefore no consciousness. Protoplasma 258(2):239–248. 10.1007/s00709-020-01550-9 32880005 10.1007/s00709-020-01550-9 PMC7907021

[CR12] Gagliano M (2018) Thus spoke the plant: a remarkable journey of groundbreaking scientific discoveries and personal encounters with plants. North Atlantic Books, Berkeley

[CR13] Galston AW (1974) The unscientific method. Bioscience 24:415–416

[CR14] Galston AW, Slayman CL (1979) The not-so-secret life of plants: in which the historical and experimental myths about emotional communication between animals and vegetable are put to rest. Am Sci 67:337–344

[CR15] Gagliano M, Vyazovskiy VV, Borbély AA, Grimonprez M, Depczynski M (2016) Learning by association in plants. Sci Rep 6:38427. 10.1038/srep38427 10.1038/srep38427 PMC5133544 27910933

[CR16] Hill RW, Wyse GA, Anderson M, Anderson M (2012) Animal physiology (Vol. 2, pp. 419–454). Sinauer associates, Sunderland, MA.

[CR17] Lev-Yadun S (2015) Suicide is painless, but salad is not. Trends Ecol Evol 30:304–305. 10.1016/j.tree.2015.04.008

[CR18] Mallatt J, Blatt MR, Draguhn A, Robinson DG, Taiz L (2021a) Debunking a myth: plant consciousness. Protoplasma 258(3):459–476. 10.1007/s00709-020-01579-w 33196907 10.1007/s00709-020-01579-w PMC8052213

[CR19] Mallatt J, Taiz L, Draguhn A, Blatt MR, Robinson DG (2021c) Integrated information theory does not make plant consciousness more convincing. Biochem Biophys Res Commun 564:166–169. 10.1016/j.bbrc.2021.01.022 33485631 10.1016/j.bbrc.2021.01.022

[CR20] Mallatt J, Robinson DG, Draguhn A, Blatt M R, Taiz L (2021b) Understanding plant behavior: a student perspective: response to Van Volkenburgh et al. Trends Plant Sci 26(11):1089-1090.10.1016/j.tplants.2021.02.012 10.1016/j.tplants.2021.08.014 34548214

[CR21] Mallatt J, Robinson D, Blatt MR, Draguhn A, Taiz L (2023) Plant sentience: the burden of proof. Anim Sent 33(15). 10.51291/2377-7478.1802

[CR22] Mancuso S, Viola A (2015) Brilliant green: the surprising history and science of plant intelligence. Island Press, Washington, DC

[CR23] Mancuso S (2018) The revolutionary genius of plants: a new understanding of plant intelligence and behavior. Atria Books, New York

[CR24] Miguel-Tomé S, Llinás RR (2021) Broadening the definition of a nervous system to better understand the evolution of plants and animals. Plant Signal Behav 16(10):1927562. 10.1080/15592324.2021.1927562 34120565 10.1080/15592324.2021.1927562 PMC8331040

[CR25] Pace E (1996) Obituary: Christopher Bird, 68, a best-selling author. New York Times, 5 May, p D9

[CR26] Ponkshe A, Barroso JB, Abramson CI, Calvo P (2023) A case study of learning in plants: lessons learned from pea plants. Quart J Exp Psychol 1–9. 10.1177/17470218231203078 10.1177/17470218231203078 37705453

[CR27] Raja V, Silva PL, Holghoomi R, Calvo P (2020) The dynamics of plant nutation. Sci Rep 10(1):19465. 10.1038/s41598-020-76588-z 33173160 10.1038/s41598-020-76588-z PMC7655864

[CR28] Rapoport A (1960) Fights, games, and debates. University of Michigan Press, Ann Arbor

[CR29] Rapoport A (1961) Three modes of conflict. Manage Sci 7(3):210–218

[CR30] Robinson DG, Draguhn A (2021) Plants have neither synapses nor a nervous system. J Plant Physiol 263:153467. 10.1016/j.jplph.2021.153467 34247030 10.1016/j.jplph.2021.153467

[CR31] Robinson DG, Mallatt J, Peer WA, Sourjik V, Taiz L (2024) Cell consciousness: a dissenting opinion: the cellular basis of consciousness theory lacks empirical evidence for its claims that all cells have consciousness. EMBO Rep 25(5):2162–2167. 10.1038/s44319-024-00127-4 38548972 10.1038/s44319-024-00127-4 PMC11094104

[CR32] Robinson DG, Blatt MR, Draguhn A, Taiz L, Mallatt J (2023) Plants lack the functional neurotransmitters and signaling pathways required for sentience in animals. Anim Sent 33(7). 10.51291/2377-7478.1782

[CR33] Ryan JC, Vieira P, Gagliano M (eds) (2021) The mind of plants: narratives of vegetal intelligence. Synergetic Press, Santa Fe

[CR34] Schlanger Z (2024) The light eaters: how the unseen world of plant intelligence offers a new understanding of life on earth. HarperCollins, New York

[CR35] Segundo-Ortin M, Calvo P (2023) Plant sentience? Between romanticism and denial: science. Anim Sent 33(1). 10.51291/2377-7478.1772

[CR36] Simard SW (2018) Mycorrhizal networks facilitate tree communication, learning, and memory. In: Baluška F, Gagliano M, Witzany G (eds) Memory and learning in plants. Springer, Cham, pp 191–213

[CR37] Simard SW (2022) Finding the mother tree: discovering the wisdom of the forest. Alfred A, Knopf, New York

[CR38] Taiz L, Alkon D, Draguhn A, Murphy A, Blatt M, Thiel G, Robinson DG (2020) Reply to Trewavas et al. and Calvo and Trewavas. Trends Plant Sci 25(3):218–220. 10.1016/j.tplants.2019.12.020 31926764 10.1016/j.tplants.2019.12.020

[CR39] Taiz L, Alkon D, Draguhn A, Murphy A, Blatt M, Hawes C, ... Robinson D G (2019) Plants neither possess nor require consciousness. Trends in plant science 24(8):677–687. 10.1016/j.tplants.2019.05.008 10.1016/j.tplants.2019.05.008 31279732

[CR40] Tang SK, Marshall WF (2018) Cell learning. Curr Biol 28:R1180–R1184. 10.1016/j.cub.2018.09.015 10.1016/j.cub.2018.09.015 PMC9673188 30352182

[CR41] Tompkins P, Bird C (1973) The secret life of plants. Harper and Row, New York

[CR42] Trewavas A (2014) Plant behavior and intelligence. Oxford University Press, Oxford

[CR43] Wohlleben P (2016) The hidden life of trees: what they feel, how they communicate. Greystone Books, Vancouver, Berkeley

